# Community-Based Healthcare Programs Sustainability Impact on the Sustainability of Host Organizations: A Structural Equation Modeling Analysis

**DOI:** 10.3390/ijerph16204035

**Published:** 2019-10-21

**Authors:** Sebastian Ion Ceptureanu, Eduard Gabriel Ceptureanu

**Affiliations:** Department of Management, The Bucharest University of Economic Studies, 010374 Bucharest, Romania; eduard.ceptureanu@man.ase.ro

**Keywords:** community-based program, host organization, sustainability, Structural Equation Modeling

## Abstract

The sustainability of community-based programs represents a major focus of the literature on community-based interventions in the last few decades. However, without sustainable host organizations to effectively implement them, many are prone to failure. This paper analyzes the influence of the sustainability factors of healthcare community-based programs on the host organization’s sustainability. Based on a sample of 11 community-based healthcare programs and 401 respondents and using structural equation modeling, the study investigated if program specific, organization specific, and community specific factors are indeed measures of community-based programs’ sustainability, if social and economic dimensions are measures of host organization sustainability, and if the sustainability of the community-based program influences thee host organization’s sustainability. The results confirmed all three research hypothesis. The main contribution of the paper is to demonstrate a direct relationship between the sustainability of community-based programs and the overall sustainability of the organizations implementing them.

## 1. Introduction

Community-based programs (CbPs) have been increasingly present in the literature in the last few decades [1,2,3,4] due to their impact on local communities such as through an efficient, transparent, and more equitable use of local resources [5,6,7,8] or improving the standards of living in the targeted communities [9,10,11,12]. 

The sustainability of CbPs is also important for the host organization, since it may also affect their sustainability [13]. An important share of the CbPs terminates unexpectedly in their very first few years after initiation, usually after the initial support is finished [1], thus failing to provide significant results for the communities. Unexpected termination of CbPs leads to negative effects for the targeted communities, since they may lose trust in similar such initiatives in the future, but also for the host organizations, since the initiation costs for a community-based program are usually high [14]. However, sustainability is rarely included in CbP planning [15], with items like program immediate outcomes prevailing instead [16,17,18]. 

Various studies have analyzed CbP sustainability by focusing on its continuation after a targeted intervention has been terminated [1,3,4,5]. In this case, activities are implemented according to the program goals [18,19]. Others have argued that CbPs may be considered sustainable only after their institutionalization in relevant organizations [20,21]. 

While the literature presenting the sustainability of community-based programs and organizational sustainability is extensive, the relationship between CbP sustainability and sustainability of the host organization has rarely been analyzed. This constitutes the rationale of this study and the research gap that it plans to fill.

The rest of this paper is organized as follows. Section 1 provides the rationale of the study and presents the research problem. Section 2 presents the literature review divided into two subsections, each one providing the theoretical background of *CbP sustainability* and *Host organization sustainability*, the research hypothesis and the variables for each construct. Section 3 presents the material and methods employed in analyzing the research problem. Section 4 present the results and discussion. Section 5 discusses the implications of the findings and highlights our conclusions.

## 2. Literature Review

### 2.1. Community-Based Program Sustainability

Community-based programs represent social interventions seeking to change social structures and institutions in the community. Their sustainability is inconsistently defined in the literature [22], especially due to the diverse landscape of CbPs and the multitude of stakeholders involved in their planning, implementation, and assessment [10]. Community-based program sustainability is considered in the literature in terms of the program continuing in its entirety [1,2], the continuity of specific program components [22], the improvement of community capacity [13,20,21], or the capacity of the CbP to continuously respond to community problems [23], which is proof of the heterogeneity of existing approaches.

CbPs intrinsically rely on community-based approaches by creating partnerships throughout their implementation [4]. Therefore, their sustainability depends not only on themselves and the capabilities of the host organizations to implement them, but also on the stakeholders [24]. Moreover, CbPs not only require the targeted community’s acceptance, but also community involvement. Thus, to be successful in the long term, CbPs have to incorporate the targeted community’s needs and accept stakeholder involvement [25] by improving program accountability [26]. As CbPs must consider and account for the targeted communities’ cultural identities to be sustainable [27], without socio-cultural acceptability, their chances of being sustainable will be low, hindered during implementation by a lack of trust or rejection [3].

Unfortunately, a comprehensive overview of the *CbP sustainability* factors does not exist [23,28,29]. Various investigations have used different and sometimes divergent approaches to identify different *CbPs sustainability* factors, therefore making it difficult to assess the relevance and relative importance of each one. There are general approaches, emphasizing broader categories of factors such as the importance of key people involved in program implementation [13,30,31], the organizational setting of the host organization [11,13,14,30,31,32,33], the social and political environment of the community-based program [11,13,17,32,33], and the financial resources required or available for program implementation [11,14,32]. Other authors [34,35,36,37,38,39,40,41,42,43,44,45,46,47,48,49,50] have argued that CbP sustainability is determined by specific factors such as program champions, stakeholder capabilities, program leadership, effective collaboration with the targeted community, or community support for the CbP.

This study used a previously tested model [4] comprising of three main factors: (1) related to the program itself; (2) related to the host organization, and (3) related to the community. The model was proven to be suitable for the Romanian context [4] (Table 1).

Therefore, we hypothesized that

**Hypothesis** **1.** *Program specific, organizational specific, and community specific constructs are measures of CbP sustainability.*

### 2.2. Host Organization Sustainability

We considered the sustainability of non-profits in discussing organizational sustainability. This ensures a comprehensive range for organizations that may act as host organizations for community-based programs. Sustainability for non-profits means that they continue to fulfill their mission and satisfy the key stakeholders’ requirements, regardless of the difficulties encountered [51].

For non-profits, the donors assume a central role because they are critical actors in providing the revenue flow [51]. Non-profits also have to cope with the significant volatility of their revenue sources, making multiple stakeholder management more complex. More stakeholders mean that non-profits have to find a fine balance between money and their mission [52,53]. On the other hand, non-profits have a broader range of mechanisms to ensure a flow of resources for support [54] by acquiring funds through governmental support and private donations, commercially generated revenues, fundraising and donations, cross sector partnerships, or volunteerism [55,56,57,58,59,60,61].

There are different perspectives on the sustainability of non-profits. One approach focuses on the financial viability [62,63,64,65,66,67,68], probably the most common in the literature and an important factor in considering the economic dimension in our model. 

Another approach, focused on organization [69], still considers funding as important, but has a more balanced approach, bringing forward items like leadership, program development and management, or the quality of resources. A development of this approach [70,71] links non-profit sustainability to several factors, namely strategy, culture, operations, people, and the business model. 

In parallel with these approaches, the social mission of non-profits is discussed in the literature in relation with sustainability, since many argue that the ultimate goal of non-profits is to increase social value [72,73,74,75]. This provided the rationale for part of the second dimension of the *Host organization sustainability* factor, the social one.

Finally, one last approach, a result of increased marketization of the non-profit sector, focuses on the implementation of business principles in non-profits [76]. To survive, non-profits are becoming more entrepreneurial [77], become more adept with innovative practices and improve attitude toward change [53], or begin redefining their mission [78,79]. This provided the rationale for the second dimension of the model, namely, the social dimension. 

Again, for host organization sustainability construct, we used a previous tested model [80], comprising Social Dimension with the items *Mission achievement, Public image, Risk acceptance, Initiative, Attitude toward change* and *Entrepreneurial approach*, and Economic Dimension with the items *Reporting compliance, Revenue diversification, Financial planning* and *Stability of revenue* [80,81,82,83,84,85,86,87,88,89,90,91,92] (Table 2):

Therefore, we hypothesized that

**Hypothesis** **2.** *Social dimension and economic dimension are measures of organizational sustainability.*

In the end, one more assumption was made:

**Hypothesis** **3.** *CbP sustainability positively and significantly influences host organization sustainability.*

## 3. Materials and Methods

For sampling, a purposive sampling strategy was used. Homoscedasticity was checked through Levene’s test [93]. Tests on the homogeneity of variances indicted that the sample across all of the control variables were homogeneous (indicated by Levene statistic > 0.05) on all control variables: host organization target (urban/rural), age, and size. The respondents were associated with specific community-based healthcare programs. Thus, N_1_ (N_1_ = 11) represents the number of host organizations surveyed, each one implementing one community-based healthcare program, while N_2_ represents the number of respondents associated with a specific healthcare CbP. These include members of the community, employees, and volunteers of host organizations engaged in community-based program implementation. No respondents were allowed to provide answers for more than one CbP. The sample composition is presented in Table 3.

Most of the host organizations operating in urban areas (64%) had more than five years of expertise in implementing community-based programs (73%) and were large (82%). In terms of support provided, two CbPs were supported by non-profits, while most were supported by town hall or county authorities. Healthcare services included heart disease prevention and diabetes (equally 18%), smoking prevention (9%), healthy nutrition (9%), and mixed (46%). The questionnaires were collected over a period of eight months, from February to September 2019. No ethical issues arose during data collection, while the respondents were assured about the confidentiality of their answers. In terms of permits, since the questionnaire included only non-medical topics, no permits were necessary or required. 

Exploratory factor analysis (EFA) followed by confirmatory factor analysis (CFA) were used for validation [92,93]. Factors with eigenvalues greater than 1 were retained [94,95]. Items with factor loadings larger than 0.40 were retained [96]. For EFA with individual factors, the identified number of items had an overall Kaiser–Meyer–Olkin (KMO) value above 7.5 [97]. The conceptual mode and research hypothesis were tested using SEM-Amos (SPSS, version 22) [98]. Orthogonal varimax with Kaiser normalization was used on all items. Path analysis further supported the findings established through EFA [99]. Regarding multicollinearity, to rule it out, variance inflationary factor (VIF) scores were checked. Table 4 shows the results with all items below the recommended threshold of 2.5 [93,100].

In terms of reliability, Cronbach’s alpha was used. The reliability test of the data for the structure of both *CbP Sustainability* and *Host organization sustainability* factors showed good internal consistency, higher the recommended threshold of 0.7 [101] (Table 5 and Table 6).

EFA was used to measure the shared variance of factors and to identify the relationships between items [102]. No prior assumptions were made about the relationships between the factors. The items with eigenvalues >1 were considered during the EFA, while the minimum threshold of 0.4 was taken to retain the items loading on to their respective factors. After EFA, principal component analysis (PCA) with varimax rotation was performed. Table 7 shows the rotated component matrix for all of the items retained. Considering the threshold value, the loadings indicate that the factor structure is valid.

To analyze the relationship between the variables, the Pearson product moment correlation test was used. All correlation values showed positive correlations (Table 8).

## 4. Results and Discussion

The SEM model shows that all of the factors were significant (Table 9). All three of the *CbP Sustainability* factors constructed (program specific, organizational specific, and community specific) (*p* < 0.001) were found to be significant measures of *CbP Sustainability*. Among the individual factors, the program specific construct was a significant measure of *CbP Sustainability* with a high and positive coefficient value of 0.703, followed by organizational specific with a coefficient value of 0.682, and community specific with a coefficient value of 0.533. Therefore,

**Hypothesis** **1.** Program specific, organizational specific, and community specific constructs are measures of CbP Sustainability.

is supported. 

Similarly, the social dimension and economic dimension were found to be significant measures of *Host organization sustainability*. The highest value of the *Host organization sustainability* measure was evident in the economic dimension, with a path coefficient of 0.612, while the social dimension had a path coefficient value of 0.368, *p* < 0.001. Therefore,

**Hypothesis** **2.** Social dimension and economic dimension are measures of organizational sustainability.

is supported.

Finally, *CbP Sustainability* was found to significantly and positively influence *Host organization sustainability*. The path coefficient values showed that the *CbP Sustainability* significantly and positively influenced *Host organization sustainability*, showing a path coefficient value of 0.742, (*p* < 0.001), which supports the research

**Hypothesis** **3.** *CbP sustainability positively and significantly influences Host organization sustainability.*

In the literature, the relationship between the constructs had not been tested. The results of the SEM indicate that *CbP Sustainability* factors not only influence *Host organization sustainability*, but is also an antecedent of it.

The absolute fit indices for the model were good with a CMIN/DF of 1.545, GFI of 0.953, AGFI of 0.853, and CFI value in the model of 0.912. The RMSEA value in this model was 0.049.

Therefore, all research hypotheses were confirmed. Table 10 summarizes the results.

## 5. Conclusions

The literature on healthcare community-based programs or initiatives is very diverse [103]. Most of the papers have described and analyzed specific factors for the success or continuity of CbPs [103,104,105]. 

The findings of this study bring new insights into the relationship between the sustainability of community-based programs and the sustainability of the host organizations, a field of research scarcely covered by the literature to date. One of the key premises of the research was that the three factors—program specific, organizational specific, and community specific—are significant for *CbP Sustainability*. This was found to be true. 

Throughout this paper, healthcare was the setting and not the focus of the paper. In fact, it is very difficult to target broader types of healthcare CbPs, since they are both very diverse (targeted community, local conditions, funding entities, stakeholders, and organizational setting to implement the program) and stakeholders may assess their results differently. 

Another issue regards their sustainability. To make matters worse, no agreed-upon definition exists for the term healthcare program sustainability [106]. While in the case of organizations (both for and non-profit), these are mostly responsible for their long-term sustainability, CbPs in most cases rely more on external factors. Sometimes, sustainability is included as part of the implementation, narrowing the perspective in that way. Furthermore, factors that influence the successful initial implementation of CbPs are not necessarily the same factors that enable continued implementation. Healthcare community-based programs take place in both clinical and community settings, with interventions delivered by individual providers; other programs more often occur in a community setting such as community partnerships. This is why we had to consider, in our analysis, factors related to the host organizations, the programs themselves, and the communities where CbPs have been implemented. On the other hand, the sustainability factors had to be general, since each type of healthcare program has its own clinical setting.

In this research, considering all the factors, program specific was the most prominent measure of the *CbP Sustainability* construct with a path coefficient value of 0.703, *p* < 0.001, in the SEM model. The result indicates that the program specific is a key factor that enables *CbP Sustainability*. CbP sustainability may be compromised without first considering the program itself. Program coordinator competence, program transparency, involvement of qualified staff in all stages of the program, the ability to address changes of community needs, making resources, and especially financial resources, available, a coherent framework, capability to document program success to make it visible for stakeholders, the ability of the program to adapt and evolve from the original plan, according to changing circumstances, and to align to the reporting requirements of stakeholders, accepting and integrating champions, have a profound integration with the host organization at all levels, having a congruent mission, capability to identify and integrate community needs and resources, but also stakeholder management by adapting to their policies and regulations, are elements that have to be considered by CbP initiators to increase the chances for success.

The organizational specific construct was found to influence *CbP Sustainability*, with a path coefficient value of 0.682 and *p* < 0.001. The host organization’s senior management capacity to establish organizational goals congruent with the CbP, its procedures, and mechanisms, the ability to adapt its internal regulations and procedures to various requirements, its capacity to initiate and maintain relations with multiple partners, but also specific organizational actions targeting sustainability may influence community-based program sustainability.

While various scholars consider the community more than other factors as critical for CbP sustainability, in this study, community specific variables, even though important, placed only third, with a path coefficient value of 0.16 and *p* < 0.001. Although the results were significant, the path coefficient value was lower when compared to other dimensions, particularly those that were program specific. This is an indication that for many, the success and continuation of a community-based program relies more on the features of the program itself and the support of the host organization than the community where it is implemented. Since usually the communities targeted by CbPs are poor, this is understandable. Still, in designing an effective CbP, elements like community involvement in CbP planning and implementation, community relations with various public or private bodies and agencies, and involvement in providing additional resources to CbP, particularly financial contributions or target group(s) availability for CbP are important.

In terms of *Host organizations sustainability*, the economic dimension is more important than the social one (0.612 versus 0.368). The analysis of the findings also showed that *CbP Sustainability* factors positively impacted on the *Host organization sustainability*, which was measured through two dimensions: social and economic. The complete SEM model showed a path coefficient value of 0.742 with *p* < 0.001, indicating significant impact of *CbP Sustainability* on *Host organization sustainability*. Since the entire conceptual model was studied as an input–output mode, it was hypothesized that *CbP Sustainability* factors are input measures that impact on *Host organization sustainability*. 

In terms of future research, there are several avenues which have to be explored: (1) A thorough analysis regarding the extent of each *CbP Sustainability* variable on specific factors of the host organization’s sustainability has to be undertaken; and (2) the development of frameworks and conceptual models regarding the factors likely to affect the sustainability of healthcare. These frameworks have to cover all three major stakeholders of the CbPs: the community, funding provider, and host organization. This paper focused on host organizations and considered their perspective, while neglecting to some extent the community and fund providers. Perhaps combining frameworks developed from the perspective of all stakeholders will make the assessment simpler and consider healthcare CbPs. (3) Sustainability has to be analyzed both in terms of outcomes and processes. There seems to be, in the literature, two streams of healthcare CbPs sustainability research: one focused on the phases of community-based program operationalization that focuses on various sustainability issues during planning, implementation etc., while the second stream focuses on the outcomes, putting aside various phases in implementation.

## Figures and Tables

**Table 1:** *Community-based program sustainability* construct.

Variables	Description	References
**I. Program Specific**		
*Program coordinator competence*	CbP coordinator ability effectively run the program	Akerlund, 2000; Hanson & Salmoni, 2011; Montemurro et al., 2014; Mancini & Marek, 2004; Ceptureanu et al., 2018
*Program transparency*	CbP capability to inform stakeholders of its results and outcomes, using suitable methods	O’Loughlin et al., 1998; Holder & Moore, 2000; Savaya et al., 2008; Ceptureanu et al., 2018
*Qualified HR involvement*	use of qualified staff in all stages of CbP	O’Loughlin et al., 1998; Holder & Moore, 2000; Mancini & Marek, 2004; Estabrooks et al., 2011; Hanson & Salmoni, 2011; Ceptureanu et al., 2018
*Program responsivity*	CbP ability to address changes of community needs	Akerlund, 2000; Holder & Moore, 2000; Mancini & Marek, 2004; Ceptureanu et al., 2018
*Program funding*	CbP availability of financial resources	Light, 1998; Shediac-Rizkallah & Bone, 1998; Akerlund, 2000; Holder & Moore, 2000; Goodson et al., 2001; Steadman et al., 2002; Mancini & Marek, 2004; Scheirer, 2005; Stevens & Peikes, 2006; Estabrooks et al., 2011; Oino et al., 2015; Ceptureanu et al., 2018
*Program theory*	CbP coherent framework	Steadman et al., 2002; Weiss et al., 2002; Savaya et al., 2008; Ceptureanu et al., 2018
*Program effectiveness*	CbP capability to document its success and make it visible for stakeholders	Shediac-Rizkallah & Bone, 1998; Pentz, 2000; Steadman et al., 2002; Mancini & Marek, 2004; Padgett et al., 2005; Ceptureanu et al., 2018
*Program flexibility*	CbP ability to adapt and evolve from the original plan, according to changing circumstances	O’Loughlin et al., 1998; Scheirer, 2005; Savaya et al., 2008; Ceptureanu et al., 2018
*Program evaluation*	CbP capability to align to the reporting requirements of stakeholders	Weiss et al., 2002; Johnson et al., 2004; Savaya et al., 2008; Ceptureanu et al., 2018
*Program champions*	individuals related to CbP promoting it in the community	Smith et al., 1993; Shediac-Rizkallah & Bone, 1998; O’Loughlin et al., 1998; Holder & Moore, 2000; Goodson et al., 2001; Steadman et al., 2002; Mancini & Marek, 2004; Scheirer, 2005; Savaya et al., 2008; Ceptureanu et al., 2018
*Program integration*	CbP of dependence to the host organization in terms of mission and strategy	Smith et al., 1993; Shediac-Rizkallah & Bone, 1998; O’Loughlin et al., 1998; Goodson et al., 2001; Johnson et al., 2004; Padgett et al., 2005; Ceptureanu et al., 2018
*Understanding the community*	CbP capability to identify and integrate community needs and resources	Shediac-Rizkallah & Bone, 1998; Holder & Moore, 2000; Pentz, 2000; Mancini et al., 2003; Mancini & Marek, 2004; Oino et al., 2015; Ceptureanu et al., 2018
*Political legitimation*	CbP adaptation to the policies and regulations of relevant stakeholders	Pentz, 2000; Pluye et al., 2004; Sarriot et al., 2004; Scheirer, 2005; Ceptureanu et al., 2018
**II. Organizational Specific**		
*Leadership*	host organization senior management capacity to establish organizational goals congruent with CbP	Shediac-Rizkallah & Bone, 1998; Akerlund, 2000; LaFond et al., 2002; Sarriot et al., 2004; Mancini & Marek, 2004; Nu’Man et al., 2007; Jacobs et al., 2007; Argaw et al., 2007; Gruen et al., 2008; Ceptureanu et al., 2018
*Organizational system*	host organization procedures and mechanisms (HR and financing), which may impact CbP outcomes	Shediac-Rizkallah & Bone, 1998; LaFond et al., 2002; Mancini & Marek, 2004; Sarriot et al., 2004; Johnson et al., 2004; Beery et al., 2005; Robinson et al., 2005; Jacobs et al., 2007; Nu’Man et al., 2007; Gruen et al., 2008; Estabrooks et al., 2011; Mijnarends et al., 2011; Ceptureanu et al., 2018;
*Organizational stability*	host organization ability to adapt its internal regulations and procedures, which may impact CbP outcomes	Shediac-Rizkallah & Bone, 1998; Goodson et al., 2001; LaFond et al., 2002; Sarriot et al., 2004; Johnson et al., 2004; Pluye et al., 2005; Argaw et al., 2007; Nu’Man et al., 2007; Jacobs et al., 2007; Gruen et al., 2008; Ceptureanu et al., 2018;
*Partnering*	host organization capacity to initiate and maintain relations with multiple partners, which may impact CbP outcomes	LaFond et al., 2002; Sarriot et al., 2004; Nu’Man et al., 2007; Hanson & Salmoni, 2011; Montemurro et al., 2014; Oino et al., 2015; Ceptureanu et al., 2018
*Specific sustainability actions*	host organization actions specifically targeting sustainability, which may impact CbP outcomes	Johnson et al., 2004; Beery et al., 2005; Robinson et al., 2005; Ceptureanu et al., 2018
**III. Community Specific**		
*Community participation*	targeted community involvement in CbP planning and implementation	Sarriot et al., 2004; Sarriot et al., 2004; Jacobs et al., 2007; Argaw et al., 2007; Gruen et al., 2008; Oino et al., 2015; Ceptureanu et al., 2018
*Community political context*	targeted community relations with various public or private bodies and agencies, which may impact CbP outcomes	Shediac-Rizkallah & Bone, 1998; Weiss et al., 2002; Sarriot et al., 2004; Sarriot et al., 2004; Jacobs et al., 2007; Gruen et al., 2008; Estabrooks et al., 2011; Mijnarends et al., 2011; Ceptureanu et al., 2018
*Community support*	targeted community involvement in providing additional resources to CbP, particularly financial contributions	Sarriot et al., 2004; Sarriot et al., 2004; Jacobs et al., 2007; Gruen et al., 2008; Montemurro et al., 2014; Ceptureanu et al., 2018
*Community capacity*	target group(s) availability for CbP from targeted community	Sarriot et al., 2004; Sarriot et al., 2004; Jacobs et al., 2007; Gruen et al., 2008; Hanson & Salmoni, 2011; Hacker et al., 2012; Montemurro et al., 2014; Oino et al., 2015; Ceptureanu et al., 2018

**Table 2:** *Host organization sustainability* construct.

**I. Social Dimension**		
*Mission achievement*	Host organization degree of achievement of its mission	Shediac-Rizkallah & Bone, 1998; Prahalad, 2004; Gray & Stites, 2013; Ceptureanu et al., 2017
*Public image*	Host organization image for stakeholders	Helmig et al., 2004; Jegers & Lapsley, 2004; Ceptureanu et al., 2017; Ceptureanu et al., 2018
*Risk acceptance*	Host organization willingness to accept risks	Thompson et al., 2000; Alvord et al., 2004; Turner & Martin, 2005; Peredo & McLean, 2006; Mair & Marti, 2006; Nicholls, 2006; Austin et al., 2006; Ceptureanu et al., 2017
*Initiative*	Host organization availability to get involved in new activities and initiatives	Sharir & Lerner, 2006; Nicholls, 2006; Ceptureanu et al., 2017
*Attitude toward change*	Host organization willingness to implement new processes and procedures	Alvord et al., 2004; Parsons & Broadbridge, 2004; Ceptureanu et al., 2017
*Entrepreneurial approach*	Host organization availability to target new beneficiaries	Turner & Martin, 2005; Austin et al., 2006; Iwu et al., 2015; Ceptureanu et al., 2017
**II. Economic Dimension**		
*Reporting compliance*	Host organization compliance with specific stakeholders rules and requirements in terms of reporting	Zietlow et al., 2007; McLaughlin, 2009; Coe, 2011; Murtaza, 2012; Weikart et al., 2013; Prentice, 2016; Ceptureanu et al., 2017
*Revenue diversification*	Host organization number of sources of revenue	Greenlee & Trussel, 2000; Keating et al., 2005; Hodge & Piccolo, 2005; Prentice, 2016; Ceptureanu et al., 2017
*Financial planning*	Host organization capability to implement adequate financial planning	Keating et al., 2005; Zietlow et al., 2007; McLaughlin, 2009; Coe, 2011; Weikart et al., 2013; Prentice, 2016; Ceptureanu et al., 2017
*Stability of revenue*	Host organization financial result perspective	Hodge & Piccolo, 2005; Keating et al., 2005; Zietlow et al., 2007; McLaughlin, 2009; Prentice, 2016; Ceptureanu et al., 2017

**Table 3:** Sample composition.

Criteria	Description	Number of Host Organizations (N_1_ = 11)	Associated Respondents (N_2_ = 401)
Host organization target (area of operations)	urban	7	257
	rural	4	144
Host organization age (no. of years since establishment)	<5	3	112
	>5	8	289
Host organization size (no. of employees, excluding volunteers)	<10	2	330
	>10	9	71
Type of support	Non-profit support	2	43
	Local support	6	96
	County support	3	262
Target	Smoking prevention	1	27
	Diabetes	2	52
	Heart diseases prevention	2	83
	Mixed	5	203
	Healthy nutrition	1	36

**Table 4:** Multicollinearity results.

Coefficients								
Model		Unstandardized Coefficients		Standardized Coefficients	t	Sig.	Collinearity Statistics	
		B	Std. Error				Tolerance	VIF
1	(Constant)	−0.298	0.788	−0.378	0.706			
	**Program Specific**							
	*Coordinator competence*	0.269	0.052	0.259	5.190	0.000	0.675	1.481
	*Transparency*	0.210	0.056	0.195	3.754	0.000	0.626	1.597
	*Qualified HR involvement*	0.208	0.050	0.206	4.152	0.000	0.682	1.466
	*Responsivity*	0.144	0.054	0.130	2.658	0.008	0.708	1.412
	*Program funding*	0.025	0.055	0.022	0.455	0.650	0.687	1.455
	*Program theory*	0.115	0.056	0.107	2.070	0.039	0.625	1.600
	*Program effectiveness*	−0.068	0.056	−0.068	−1.206	0.228	0.534	1.874
	*Program flexibility*	0.012	0.054	0.012	0.230	0.818	0.657	1.523
	*Program evaluation*	0.102	0.056	0.091	1.832	0.068	0.677	1.478
	*Program champions*	0.026	0.055	0.024	0.479	0.632	0.663	1.508
	*Program integration with the host organization*	−0.077	0.065	−0.057	−1.185	0.237	0.720	1.389
	*Understanding the community*	0.181	0.060	0.156	3.045	0.003	0.642	1.558
	*Political legitimation*	−0.006	0.055	−0.006	−0.115	0.908	0.662	1.512
	**Organizational Specific**							
	*Leadership*	−0.036	0.065	−0.028	−0.548	0.584	0.624	1.603
	*Organizational system*	−0.164	0.062	−0.136	−2.647	0.008	0.639	1.564
	*Organizational stability*	0.085	0.060	0.078	1.410	0.159	0.550	1.819
	*Partnering*	0.093	0.054	0.085	1.726	0.085	0.696	1.437
	*Specific sustainability actions*	0.017	0.056	0.016	0.300	0.764	0.601	1.663
	**Community Specific**							
	*Community participation*	−0.005	0.062	−0.004	−0.084	0.933	0.624	1.604
	*Community political context*	0.025	0.069	0.021	0.361	0.718	0.512	1.955
	*Community support*	0.032	0.065	0.027	0.495	0.621	0.575	1.740
	*Community capacity*	−0.052	0.063	−0.044	−0.823	0.411	0.599	1.671
	**Social Dimension**							
	*Address social needs*	−0.033	0.067	−0.031	−0.493	0.622	0.414	2.413
	*Public image*	0.051	0.093	0.039	0.550	0.583	0.331	2.019
	*Risk acceptance*	0.023	0.096	0.015	0.237	0.813	0.406	2.462
	*Initiative*	−0.082	0.091	−0.057	−0.900	0.369	0.426	2.346
	*Attitude toward change*	0.041	0.083	0.031	0.495	0.621	0.441	2.266
	*Entrepreneurial approach*	−0.027	0.072	−0.021	−0.383	0.702	0.564	1.773
	**Economic Dimension**							
	*Reporting compliance*	−0.134	0.068	−0.107	−1.963	0.050	0.572	1.749
	*Revenue diversification*	0.004	0.057	0.003	0.066	0.948	0.873	1.146
	*Financial planning*	−0.143	0.075	−0.113	−1.901	0.058	0.477	2.095
	*Stability of revenue*	0.093	0.086	0.074	1.076	0.283	0.354	2.821

**Table 5:** Reliability of *CbP sustainability* factors.

Factors	Cronbach’s Alpha
Program specific	0.782
Organizational specific	0.729
Community specific	0.716

**Table 6:** Reliability of *Host organization sustainability* factors.

Factors	Cronbach’s Alpha
Social dimension	0.841
Economic dimension	0.795

**Table 7:** Rotated component matrix.

Rotated Component Matrix ^a^					
	Component				
	**1**	**2**	**3**	**4**	**5**
*Coordinator competence*	0.858				
*Transparency*	0.747				
*Qualified HR involvement*	0.701				
*Responsivity*	0.676				
*Program funding*	0.659				
*Program theory*	0.648				
*Program effectiveness*	0.726				
*Program flexibility*	0.731				
*Program evaluation*	0.717				
*Program champions*	0.628				
*Program integration with the host organization*	0.696				
*Understanding the community*	0.702				
*Political legitimation*	0.598				
*Leadership*		0.778			
*Organizational system*		0.716			
*Organizational stability*		0.693			
*Partnering*		0.702			
*Specific sustainability actions*		0.684			
*Community participation*			0.626		
*Community political context*			0.611		
*Community support*			0.704		
*Community capacity*			0.722		
*Address social needs*				0.763	
*Public image*				0.845	
*Risk acceptance*				0.715	
*Initiative*				0.607	
*Attitude toward change*				0.622	
*Entrepreneurial approach*				0.659	
*Reporting compliance*					0.834
*Revenue diversification*					0.817
*Financial planning*					0.793
*Stability of revenue*					0.784

Extraction method: Principal component analysis. Rotation method: Varimax with Kaiser normalization. ^a^ Rotation converged in six iterations.

**Table 8:** Correlation matrix.

	Program Specific	Organizational Specific	Community Specific	Social Dimension	Economic Dimension
Program specific	1				
Organizational specific	0.207 *	1			
Community specific	0.033	0.042	1		
Social dimension	0.451 **	0.052	0.026	1	
Economic dimension	0.409 **	0.089	0.013	0.465 **	1

* Correlation is significant at the 0.05 level (2-tailed). ** Correlation is significant at the 0.01 level (2-tailed).

**Table 9:** Standardized regression weights: (complete Structural Equation Modeling model).

			Estimate	*p* Values
Program specific	<---	*CbP Sustainability*	0.703	***
Organizational specific	<---	*CbP Sustainability*	0.682	***
Community specific	<---	*CbP Sustainability*	0.533	***
Social dimension	<---	*Host organization sustainability*	0.368	***
Economic dimension	<---	*Host organization sustainability*	0.612	***
*Host organization sustainability*	<---	*CbP Sustainability*	0.742	***
*Coordinator competence*	<---	Program specific	0.412	0.004
*Transparency*	<---	Program specific	0.674	***
*Qualified HR involvement*	<---	Program specific	0.652	***
*Responsivity*	<---	Program specific	0.625	***
*Program funding*	<---	Program specific	0.599	***
*Program theory*	<---	Program specific	0.552	***
*Program effectiveness*	<---	Program specific	0.605	***
*Program flexibility*	<---	Program specific	0.560	***
*Program evaluation*	<---	Program specific	0.602	***
*Program champions*	<---	Program specific	0.441	0.003
*Program integration with the host organization*	<---	Program specific	0.550	***
*Understanding the community*	<---	Program specific	0.642	***
*Political legitimation*	<---	Program specific	0.575	***
*Leadership*	<---	Organizational specific	0.516	***
*Organizational system*	<---	Organizational specific	0.715	***
*Organizational stability*	<---	Organizational specific	0.525	***
*Partnering*	<---	Organizational specific	0.632	***
*Specific sustainability actions*	<---	Organizational specific	0.490	***
*Community participation*	<---	Community specific	0.602	***
*Community political context*	<---	Community specific	0.761	***
*Community support*	<---	Community specific	0.667	***
*Community capacity*	<---	Community specific	0.640	***
*Address social needs*	<---	Social dimension	0.730	***
*Public image*	<---	Social dimension	0.870	***
*Risk acceptance*	<---	Social dimension	0.740	***
*Initiative*	<---	Social dimension	0.501	0.001
*Attitude toward change*	<---	Social dimension	0.637	***
*Entrepreneurial approach*	<---	Social dimension	0.679	***
*Reporting compliance*	<---	Economic dimension	0.488	***
*Revenue diversification*	<---	Economic dimension	0.502	***
*Financial planning*	<---	Economic dimension	0.778	***
*Stability of revenue*	<---	Economic dimension	0.530	***

*** Correlation is significant at the 0.001 level (2-tailed).

**Table 10:** Hypotheses accepted after data analysis.

Hypotheses	Path Coefficient	Significance	Status
H1: Program specific, Organization specific and Community specific factors are measures of *CbP Sustainability*	0.703, 0.682, 0.533,	*p* < 0.001	Supported
H2: Social dimension and Economic dimension are measures of *Host organization sustainability*	0.368, 0.612	*p* < 0.001	Supported
H3: *CbP Sustainability* positively and significantly influences *Host organization sustainability*	0.742	*p* < 0.001	Supported
